# Multi-Functional Alginate Lyase *AlgVR7* from *Vibrio rumoiensis*: Structural Insights and Catalytic Mechanisms

**DOI:** 10.3390/md23030124

**Published:** 2025-03-13

**Authors:** Zhe Huang, Shuai Liang, Wulong Jiang, Li Wang, Yuan Wang, Hua Wang, Lianshun Wang, Yuting Cong, Yanan Lu, Guojun Yang

**Affiliations:** 1 College of Fisheries and Life Science, National Demonstration Center for Experimental Aquaculture Education, Dalian Ocean University, Ministry of Education, Dalian 116023, China; 13484851172@163.com (Z.H.);; 2 Key Laboratory of Mariculture & Stock Enhancement in North China’s Sea, Ministry of Agriculture and Rural Affairs, Dalian Ocean University, Dalian 116023, China; 3 Dalian Key Laboratory of Breeding, Reproduction and Aquaculture of Crustaceans, Dalian 116023, China

**Keywords:** multifunctional alginate lyase, alginate oligosaccharides, structural insights, catalytic mechanisms

## Abstract

In this study, we identified *AlgVR7*, a novel bifunctional alginate lyase from *Vibrio rumoiensis* and characterized its biochemical properties and substrate specificity. Sequence alignment analysis inferred the key residues K267, H162, N86, E189, and T244 for *AlgVR7* catalysis, and it is derived from the PL7 family; exhibited high activity towards sodium alginate, polyM (PM), and polyG (PG); and can also degrade polygalacturonic acid (PGA) efficiently, with the highest affinity and catalytic efficiency for the MG block of the substrate. The optimal temperature and pH for *AlgVR7* were determined to be 40 °C and pH 8, respectively. The enzyme activity of *AlgVR7* was maximum at 40 °C, 40% of the enzyme activity was retained after incubation at 60 °C for 60 min, and enzyme activity was still present after 60 min incubation. *AlgVR7* activity was stimulated by 100 Mm NaCl, indicating a halophilic nature and suitability for marine environments. Degradation products analyzed using ESI-MS revealed that the enzyme primarily produced trisaccharides and tetrasaccharides. At 40 °C and pH 8.0, its *K*_m_ values for sodium alginate, PM, and PG were 16.67 μmol, 13.12 μmol, and 22.86 μmol, respectively. Structural analysis and molecular docking studies unveiled the key catalytic residues involved in substrate recognition and interaction. Glu167 was identified as a critical residue for the PL7_5 subfamily, uniquely playing an essential role in alginate decomposition. Overall, *AlgVR7* exhibits great potential as a powerful bifunctional enzyme for the efficient preparation of alginate oligosaccharides, with promising applications in biotechnology and industrial fields.

## 1. Introduction

Alginate, consisting of α-L-guluronic acid and β-D-mannuronic acid linked by β-1, 4 glycosidic bonds, is a naturally occurring polymer acid polysaccharide. It is primarily composed of poly guluronic acid (polyG), poly mannuronic acid (polyM), and alginate (polyMG). Derived mainly from macroalgae such as kelp, sargassum, giant algae, and bubble algae within the brown algae phylum, alginate constitutes 22~44% of their dry matter [1].

Alginate oligosaccharides (AOS) are depolymerization byproducts of alginate generated either enzymatically by alginate lyase (Alys) or through physicochemical methods. These byproducts have recently gained significant attention due to their diverse applications in food, agriculture, and pharmaceutical industries [2]. AOS utilization in frozen water products may potentially enhance the efficient removal of crystalline water, consequently slowing protein degradation in these products [3]. Furthermore, AOS can stimulate cytokine production as well as regulate blood sugar and lipid levels [4]. As a result, the enzymatic depolymerization of alginate to produce biologically active AOS from brown algae remains a consistent area of research focus.

Alys, primarily derived from marine bacteria, are extensively used in food, medicine, feed, agriculture, and chemical industries [5]. To date, Alys have been classified into 14 polysaccharide lyase (PL) families, according to the Carbohydrate-Active enzymes (CAZY) database based on sequence and structural similarities. Specifically, they belong to the PL5, -6, -7, -8, -14, -15, -17, -18, -31, -32, -34, -36, -39, and -41 families. Moreover, Alys can be categorized into PG-specific lyases (EC 4.2.2.11), PM-specific lyases (EC 4.2.2.3), and bifunctional lyases (EC 4.2.2.) depending on their substrate specificities, including polyM, polyG, and polyMG [6]. Due to this property, Alys have been developed to determine the fine structure of alginate, such as block distributions or M (G) content in alginate [7]. Depending on the cleaving mode, Alys can be classified into exo-type lyases (EC 4.2.2.26) and endo-type lyases. Exo-type lyases catalyze at the terminal end of the polymer chain to yield monosaccharides, while endo-type lyases act within the polymer chain, producing oligosaccharides. Most characterized Alys degrades single substrates, but bifunctional Alys can simultaneously degrade polyM and polyG, generating different types of AOS, including oligoM and oligoG [8]. For example, Aly448 from the PL7 family possesses two entirely distinct binding sites for alginate, poly- polyG, and polyM substrates [9]. And most of the highly active fucoidan lytic enzymes are from the PL7 family; AlgNJ-07 characterized by Zhu from the *Serratiamarcescens* NJ-07 clone showed the highest activity, 6468.99 U/mg at pH 7.0 and 30 °C [10]. The highest activity of AlgH-I characterized by Yan from the *Marinimicrobium koreense* H1 clone under optimal conditions was 5510 U/mg [11].

Bifunctional Alys can more efficiently degrade alginate into AOS with varying degrees of polymerization, which exhibit distinct biological activities. 4-deoxy-1-erythron-5-hexoseuloseuronate acid (DEH), the primary product of exo-type Alys, is a crucial raw material for bioethanol production [12]. AOS with a degree of polymerization (DP) ranging from 2 to 10, generated by endolytic Alys, display unique biological activities. *Agarivorans* sp. L11 produces AOS with a DP of 2–5, which can inhibit the growth of human osteosarcoma MG-63 cells [13]. In a study by Yamamoto et al., AOS (DP3–DP6) were found to more effectively stimulate cytokine production in RAW264.7 macrophage cells compared to AOS (DP3–6) [14]. Therefore, producing AOS with different DPs using bifunctional Alys can minimize costs and maximize efficiency in utilization.

In this study, we successfully cloned and expressed a novel, bifunctional Alys-*AlgVR7* from *Vibrio rumoiensis*, which efficiently degraded alginate into AOS. The biochemical characterization and action pattern of *AlgVR7* were thoroughly investigated. This research is pivotal for the production of oligosaccharides and understanding the relationship between Alys structure and function using new bifunctional Alys.

## 2. Results

### 2.1. Phylogenetic Analysis and Sequence Comparison of AlgVR7

**Figure 1:**
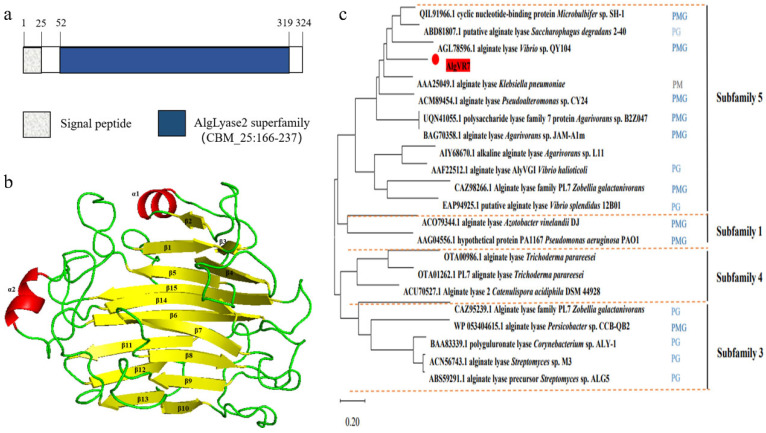
Sequence analysis of the alginate lyase *AlgVR7* from *Vibrio rumoiensis*. (**a**) Schematic domain diagram of *AlgVR7*. (**b**) The overall structure of *AlgVR7* predicted with AlphaFold. Enzymes highlighted in yellow are structure-solved 2. (**c**) Phylogenetic analysis of *AlgVR7* and other PL7 lyases from different subfamilies. The subfamily and the substrate specificity are marked in red numbers and blue annotations according to the CAZy database. *AlgVR7* is highlighted in red.

**Figure 2:**
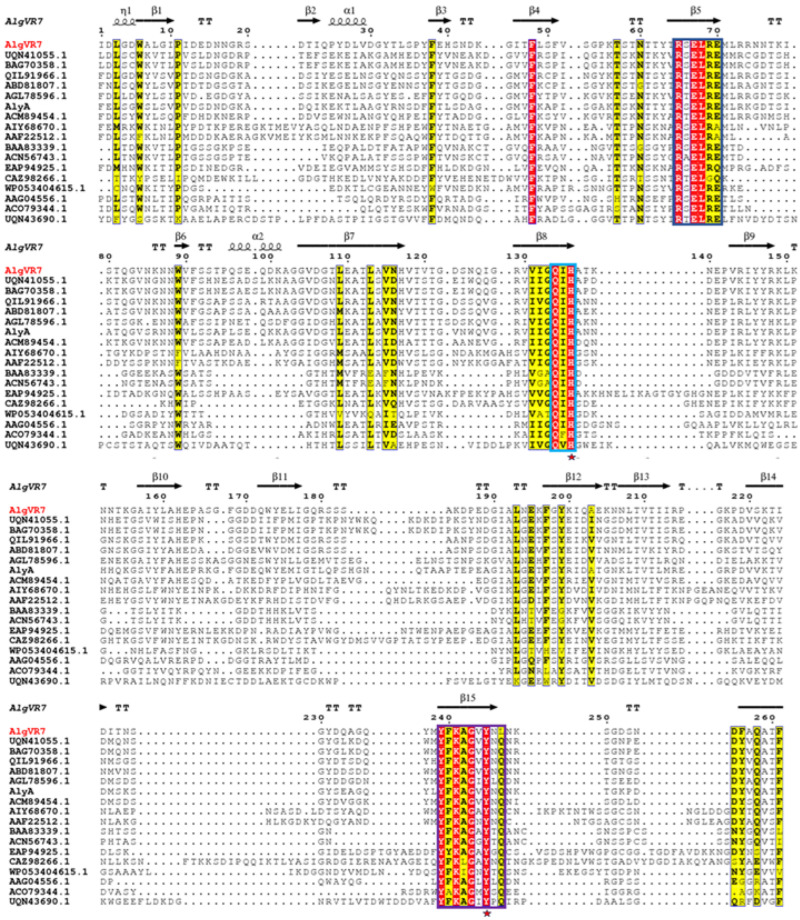
Multiple sequence alignments of *AlgVR7* and related alginate lyases of strains.

The coding region of *AlgVR7* consists of 972 bp, encoding a protein with 324 amino acids, an estimated molecular mass of 32.9 kDa, and an isoelectric point of 5.66. It is predicted that *AlgVR7* comprises a signal peptide (1–25 amino acid residues) and one conserved domain, including an N-terminal domain (NTD) ranging from Ile52 to His319 (Figure 1a). The *AlgVR7* structure forms a β-jelly roll fold with two α-helices and seven anti-parallel strands, creating two large β-sheets (Figure 1b). Phylogenetic analysis of *AlgVR7*, together with alginate lyases from seven PL families, shows that *AlgVR7* forms a distinct group within the PL7 family members (Figure 1c), suggesting that *AlgVR7* is a PL7 alginate lyase from the *Vibrio* sp. *AlgVR7*’s structure was predicted using AlphaFold 2 [15]. Part of alginate lyase from PL7 characterized so far were put together for sequence alignment. Three highly conserved regions have been identified, R*E*R, Q*H, and YFKAG*Y*Q. According to the crystal structures and sequence analyses of lyases from the PL-7 family (Figure 2).

### 2.2. Expression and Purification of AlgVR7

**Figure 3:**
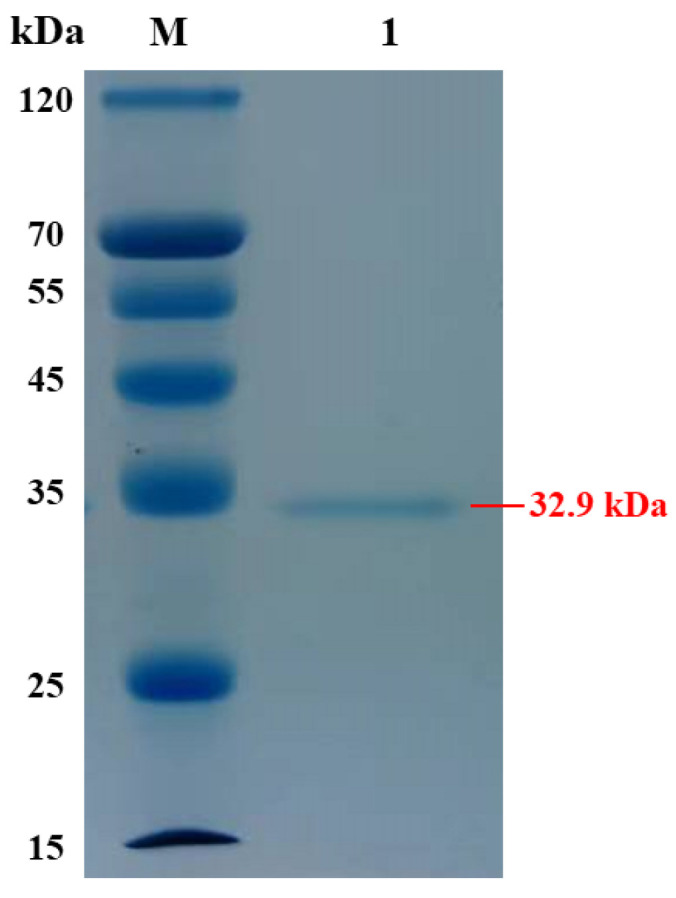
SDS-PAGE analysis of purified *AlgVR7*. Lane M: protein marker; Lane 1: purified *AlgVR7*.

The mature *AlgVR7*, without the signal peptide, was expressed in *E. coli*, and a single band was obtained after purification, as shown on a polyacrylamide gel (Figure 3). The molecular mass of *AlgVR7* was approximately 32.9 kDa, as evidenced by the protein ladder. The protein bands exhibited a single, impurity-free band suitable for subsequent experiments.

### 2.3. Substrate Specificity and Kinetic Parameters of AlgVR7

**Figure 4:**
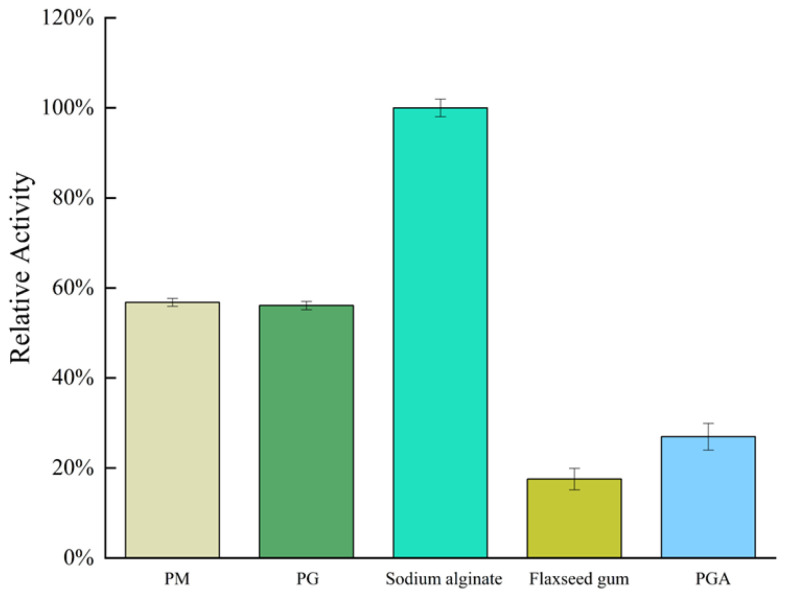
The substrate specificity of *AlgVR7*.

*AlgVR7*’s activity patterns against various substrates, such as PM, PG, sodium alginate, flaxseed gum, and PGA (Figure 4), indicate effective functioning. *AlgVR7* exhibits maximum activity on sodium alginate (100%), 60% on PM and PG, 25% on PGA, and 20% on flaxseed gum.

The activity values towards polyM, polyG, and polyMG in Table 1 indicate that *AlgVR7* is a bifunctional enzyme, displaying activities for both polyM and polyG. As shown in Table 1, using alginate, PM and PG as a substrate, the enzyme activity of *AlgVR7* was 6854.6, 3893.6, and 3845.0 U/mg, and the kinetic constants of *AlgVR7* were determined under optimal conditions, with a *V*_max_ of 186.8, 150.9, and 176.8 μmol/s; *K*_m_ of 16.6, 13.1, and 22.8 μmol; and *K*_cat_ of 16.9, 13.7, and 11.4 s^−1^.

### 2.4. Biochemical Characterization of AlgVR7

**Figure 5:**
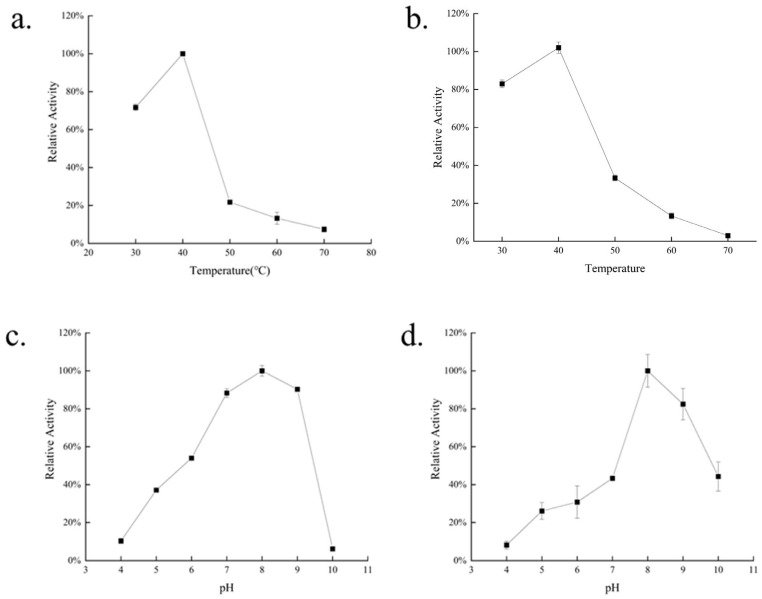
Enzymatic characterization of recombinant *AlgVR7*. (**a**) The optimal temperature of *AlgVR7*. Reactions were conducted in 50 mM PBS buffer (pH 8.0) at different temperatures for 20 min. (**b**) The effect of temperature on the stability of AlyVR7. AlyVR7 was incubated at different temperatures for 120 min, and the residual activities were measured at 40 °C and pH 8.0. (**c**) The optimal pH of *AlgVR7*. Reactions were conducted at 40 °C for 20 min in 50 mM PBS buffer over a pH range from 4.0 to 10.0. (**d**) The pH stability of *AlgVR7*. After incubating the enzyme in buffers of various pH at 4 °C for 24 h, the residual enzyme activities were measured in 50 mM PBS buffer (pH 8.0) at 40 °C. The highest activity was set to be 100%. Each value represents the mean of three replicates ± standard deviation.

Enzyme activity assays were conducted under various temperature and pH conditions to determine the optimal conditions and stability of *AlgVR7* (Figure 5). *AlgVR7* exhibited maximal activity at 40 °C and maintained over 70% of peak activity at suboptimal temperatures between 35 and 45 °C. Maximum activity occurred at pH 8. In the thermostability assay, *AlgVR7* remained relatively stable at 40 °C, while it was unstable at other temperatures. The temperature where the enzyme shows 50% activity following 120 min incubation is 47–48 °C. Incubation at 30 °C or 50 °C for 120 min led to a 20% or 65% decrease, respectively, in enzyme activity, and *AlgVR7* lost nearly all activity after 120 min at 70 °C. These results suggest that *AlgVR7* is thermally unstable. Overall, our data indicate that *AlgVR7* is a mesophilic, alkaline Alys.

In Table 2, most PL7 bifunctional alginate lyases from *Vibrio* sp. optimum temperature is 30–40, and optimum pH is 4.0–8.0. Under the same enzyme activity assay method, *AlgVR7* has the highest enzyme activity of 6854.6 U/mg.

### 2.5. Effects of Metal Ions on AlgVR7 Stability

**Figure 6:**
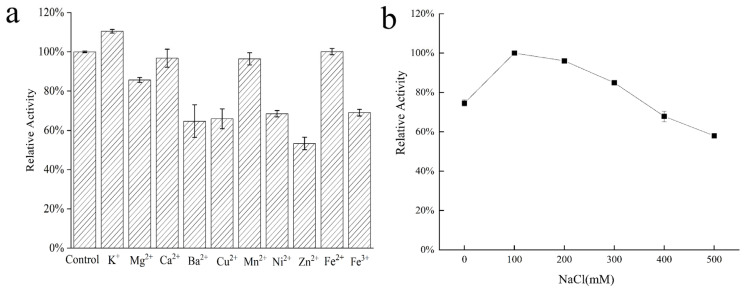
Effects of metal ions on *AlgVR7* stability. (**a**) Stability of *AlgVR7* to various metal ions. (**b**) Stability of *AlgVR7* to NaCl.

Metal ions can be essential for maintaining enzyme activity by modulating the catalytic process or enzyme structure [21,22,23]. To examine their effects on *AlgVR7* stability (Figure 6), the enzyme was incubated with various additives at 40 °C for 1 h under optimal conditions. These additives included 1 mM of metal ions, such as Na^+^, K^+^, Ca^2+^, Mg^2+^, Fe^2+^, Cu^2+^, Co^2+^, Mn^2+^, Ba^2+^, Cd^2+^, and Zn^2+^. High-valence cations may interact with residues in the active center, disrupting enzyme–substrate binding and causing distinct inhibition of enzyme activity.

### 2.6. ESI-MS Analysis of the Degradation Products

**Figure 7:**
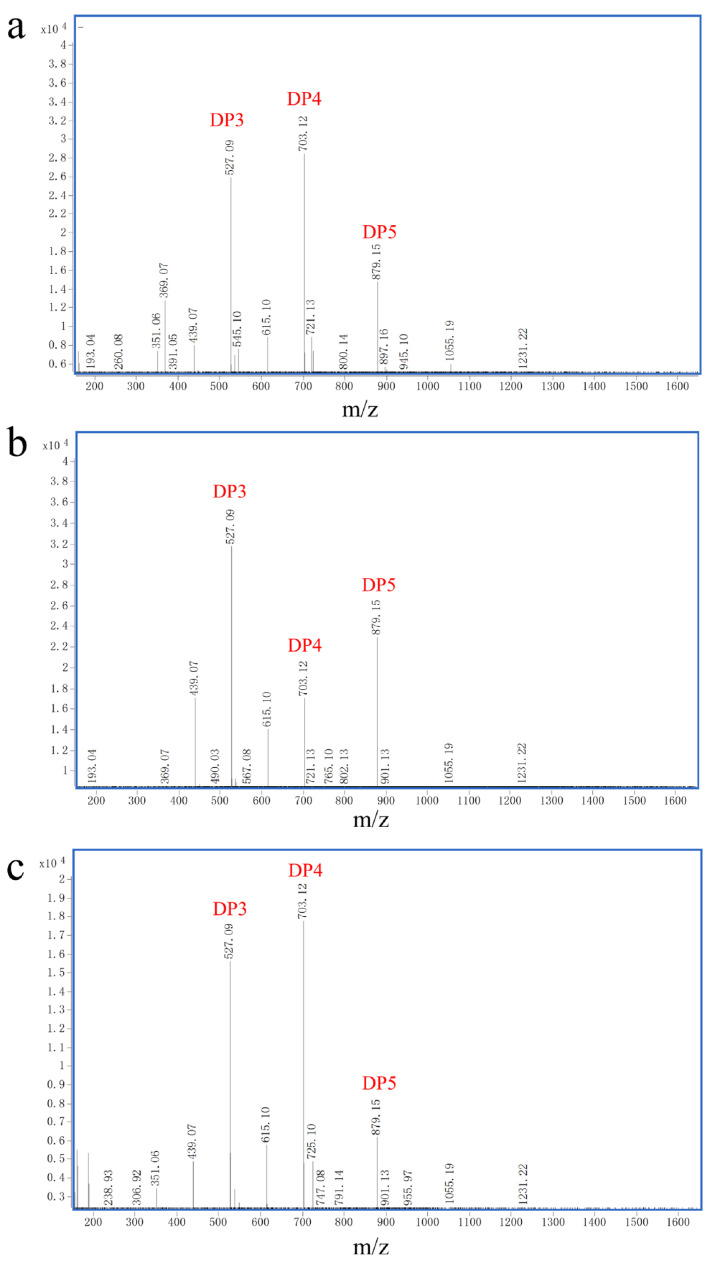
Hydrolysates from different substrates were analyzed using ESI-MS. ESI-MS analysis of hydrolysis products with (**a**) polyM used as the substrate, (**b**) polyG used as the substrate, and (**c**) sodium alginate used as the substrate.

As shown in Figure 7, the degradation products were analyzed using ESI-MS [24]. For sodium alginate, polyG, and polyM hydrolysates, the compositions of degradation products were similar, with trisaccharides and tetrasaccharides constituting a large fraction of monosaccharide types. Current commercial enzymes derived from the *Flavobacterium genus* are capable of cleaving only single substrates and obtaining 5–7 DP sugar products [25].

### 2.7. Substrate Recognizing and Molecular Docking

**Figure 8:**
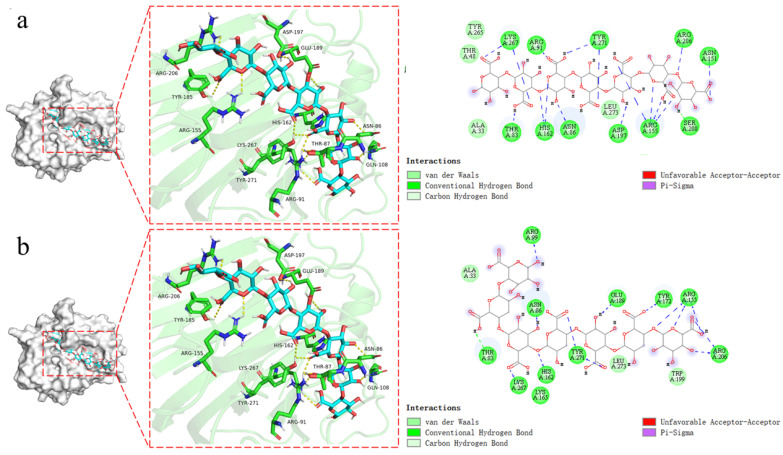
Molecular docking and clarifying catalytic residues of *AlgVR7*. (**a**) Surface representation of the binding site of PG7 in AlyVR7 (indicated by the stick, drawn in blue). Key residues (drawn in green) interacting with the PG7. Surface representation of the binding site of PG7 in AlyVR7 for 2D. (**b**) Surface representation of the binding site of PGA7 in AlyVR7 (indicated by the stick, drawn in purple). Key residues (drawn in green) interacting with thePGA7. Surface representation of the binding site of G4 in AlyVR7 for 2D.

The inner sheet forms a long tunnel to accommodate the substrate PG7 (Figure 8a) and PGA7 (Figure 8b) in the center. Key interactions between *AlgVR7* and the PM7 oligosaccharide involve recognition of the C5 carboxyl moiety on each uronic acid residue, which alternately points toward the opposite side of the substrate-binding site (K267, H162, N86, E189, and T244) (Figure 8b). Specifically, H162 and Y271 function as the catalytic base and catalytic acid, respectively. Structural alignment of alginate lyases from different PL7 subfamilies reveals that a loop called loop1, located around the active center, varies among subfamilies, with PL7_5 subfamily enzymes having a longer loop1 than other members (Figure 8b). In addition, the docking results showed that His162 and Arg155 also had catalytic effects on PGA substrates, possibly due to the inclusion of a CBM25 in the functional domain of the *AlgVR7*.

## 3. Discussion

Sequence alignment revealed that *AlgVR7* contains three typical PL7 conserved domains (R*E*R, Q*H, and YFKAG*Y*Q) responsible for substrate binding and catalysis (Figure 2), initially demonstrated in the alginate lyases VxAly7B [26] and AlgSH7 [23]. Sequence conservation analysis suggested that the conserved residues R87, H162, and Y271 in *AlgVR7* are essential for enzyme activity (Figure 2), similar to the hydrophilic residues in the counterpart AlyC3’s active center [27]. The function of CBM is more versatile, such as thermostability, enzymatic activity, substrate binding, and product distribution among different alginate lyases [28]. *AlgVR7* from *Vibrio rumoiensis* features an N-terminal CBM 25 domain, and this domain is first discovered in the PL7 family. The CBM 25 domain has an amylase function and may have a substrate specificity of α-1, 4 glycosidic bonds [29]. Additionally, a signal peptide likely aids *AlgVR7*’s localization. In PL7 alginate lyase Aly01 from *Vibrio natriegens* SK42.001, *E. coli* recognizes the signal peptide, leading to secreted protein. When the signal peptide was removed, *AlgVR7* was only expressed in the cytoplasm [30].

These findings suggest that *AlgVR7* is a bifunctional Alys acting on both polyM and polyG units of alginate molecules, making it a bifunctional lyase [31]. Michelis–Menten is an important index to measure enzyme activity. Like other PL7 enzymes, *AlgVR7*’s catalytic domain is predicted to fold as a β-jelly roll and contains a His162-Tyr271 functional motif crucial for alginate digestion [2]. Acting as a bifunctional lyase due to its ability to catalyze both polyM and polyG, *AlgVR7* from *Vibrio rumoiensis* 402 can degrade flaxseed gum and PGA with α-1, 4 glycosidic bonds due to its unique CBM25 domain, compared to other alginate lyases [32].

As shown in Table 2, *Vibrio* sp.-derived alginate lyases all have broad substrate specificity. *Vibrio rumoiensis* is a characteristic branching evolutionary species of algal polysaccharide degradation [33]. Therefore, *AlgVR7* from *Vibrio rumoiensis* 402 has a higher catalytic activity. *AlgVR7* (*K*_m_ = 16.67 μmol) showed higher sodium alginate substrate affinity than the same PL7 family of AlyA (*K*_m_ = 36.0 μmol) [34]. Then, *AlgVR7* (*K*_m_ = 2.4 mg/mL) has more substrate affinity for PM than AlgA (*K*_m_ = 28.5 mg/mL) as a bifunctional enzyme [35]. As shown in Table 2, most PL7 bifunctional alginate lyases from *Vibrio* sp. had high catalytic efficiency for one substrate; certain enzymes, such as *AlgVR7*, AlgNJ-04, and Al-gNJU-03 also had high activity towards alginate, PM, and PG. In addition, the *V*_max_ and *K*_cat_ values of *AlgVR7* for alginate, PM, and PG were greater than AlgNJ-04 and Al-gNJU-03, indicating that its strong efficient catalytic efficiency results in higher enzyme activity of *AlgVR7*.

Many Alys from marine microorganisms exhibit Na^+^-dependent activity [5]. *AlgVR7* demonstrated high stability against Na^+^ across a wide concentration range, with the most stimulative effect observed after treatment with 0.1 M Na^+^. This indicates that *AlgVR7* is halophilic and suitable for marine environments (Figure 6b). Salt is required for its alginolytic activity, as seen in other alginate lyases. In Aly1281 from *Pseudoalteromonas carrageenovora* ASY5, Na^+^ positively affected substrate binding and alginolytic activity [36]. However, the role of various metals in alginolytic activity remains largely unknown.

The verification of the composition of the degradation products was undertaken using ESI-MS (Figure 7). When sodium alginate, polyM, and polyG are used as substrates, oligomers of DP3, 4, and 5 (signals of 527.09 *m*/*z*, 703.12 *m*/*z*, and 879.15 *m*/*z*) are released as end products (Figure 7a–c). Therefore, endoactivity is in the reaction process of *AlgVR7*. It can be seen from the above results that *AlgVR7* can efficiently degrade three substrates into trisaccharides and tetrasaccharides with an endo mode. The product distribution of endo-lyase of the PL7–5 subfamily is concentrated in DP2–5. Alginate oligosaccharides (AOS) exhibit unique biological activities and health benefits, with AOS of different degrees of polymerization (DP) demonstrating varying biological activities. Dimers, trimers, and tetramers, with guluronic acid at the reducing end, can effectively induce keratinocyte proliferation when epidermal growth factor is present [37]. AOS prepared from alginate lyase from *Microbulbifer* sp. ALW1 predominantly consist of disaccharides and a few trisaccharides, displaying free radical scavenging abilities (DPPH, ABTS^+^, and hydroxyl groups) and reducing capacity [30]. AOS (DP 2–5) prepared by alginate lyase produced by *Agarivorans* sp. L11 inhibit the growth of human osteosarcoma MG-63 cells. Shorter AOS (DP 2–5) prepared from *Laminaria japonica* provide better nonspecific immunostimulatory effects on sea cucumber *Apostichopus japonicus* than highly polymerized AOS (DP 5–20), as demonstrated by higher phagocytic capacity, lysozyme activity, peroxidase activity, and total nitric acid synthase of coelomocytes [38]. *AlgVR7* possesses high affinity for natural substrates and high catalytic efficiency. Its excellent properties make *AlgVR7* a powerful tool for preparing oligomers from alginate, with promising applications in biotechnology and industrial fields. Due to *AlgVR7*’s dual function, the oligosaccharides it prepares can be of different substrate sources, which greatly strengthens its application prospects. Degradation of seaweed with blends of Cellic CTec2 and AMOR_PL7A from hot vents in the Arctic Mid-Ocean Ridge at 55 °C in seawater showed that the lyase efficiently reduces viscosity and increases glucose solubilization [39]. *AlgVR7* stably produces AOS of 3–5 DP; this means that it can make better use of the biological activity of AOS.

Further phylogenetic analysis classifies *AlgVR7* as a member of the PL7_5 subfamily. Compared to other PL7 subfamily members, some conserved amino acids at the central site (Glu167 in *AlgVR7*) vary in PL7_5 enzymes. Du et al.’s site-directed mutagenesis demonstrated that Glu392 is essential to the high alginolytic activity of VaAly2 [40]. As it is entirely conserved in the PL7_5 subfamily, Glu167 of *AlgVR7* is proposed to be a hallmark for this subfamily.

In summary, when binding to substrates, both *AlgVR7* and algal polysaccharides (PM, PG, and PMG) are capable of substrate recognition and the corresponding product. It is worth noting that due to the presence of the CBM25 domain, some residues of *AlgVR7* can also form hydrogen bonds on PGA substrates and act on α-1, 4 glycosidic bonds.

## 4. Materials and Methods

### 4.1. Materials

The strain *Vibrio rumoiensis* 402 was isolated from a sea cucumber breeding pond of Dalian Ocean University and conserved in our laboratory. Sodium alginate (M/G ratio 1) derived from *Macrosystis pyrifera* was purchased from Macklin Biochemical Co., Ltd. (Shanghai, China). PM (purity: about 99%) and PG (purity: about 99%) were purchased from Qingdao BZ Oligo Biotech Co., Ltd. (Qingdao, China). *Escherichia coli* DH5 and *E. coli* BL21 (DE3) were used as the cloning host and the protein expression host, respectively.

### 4.2. Sequence Analysis of AlgVR7

The *AlgVR7* protein sequence was came from our laboratory The sequence similarity of *AlgVR7* was analyzed using NCBI BLAST, and the protein function domains were identified using NCBI CDD and SMART (http://smart.embl-heidelberg.de/, accessed on 11 November 2023). Multiple amino acid sequence alignment was accomplished using Clustal Omega (http://www.clustal.org/omega/, accessed on 11 November 2023) and ESPript3 (https://espript.ibcp.fr/, accessed on 11 November 2023). Signal peptide was predicted using SignalP 5.0 online server. Protein phylogenetic tree construction was performed by the MEGA (v 11.0) program (Mega Limited, Auckland, New Zealand) with the neighbor-joining method.

### 4.3. Cloning of Alginate Lyase Gene AlgVR7

The strain *Vibrio rumoiensis* 402 could be found in the NCBI Genome Project (http://www.ncbi.nlm.nih.gov/genome/, accessed on 11 November 2023). The forward primer-containing restriction site *Bam H* was designed as 5-GGAATTCCATATGTACAATTTGTTGTCCGG-3, and the reverse primer containing restriction site *Xho* I was designed as 5-CCGCTCGAGCTGCTCGGTAACGGTAAC-3. The nucleotide fragments amplified via PCR were purified and ligated to pET-28a (+) expression vector (Novagen, Madison, WI, USA). The nucleotide sequence was sequenced by Beijing Genomics Institute Co., Ltd. (Beijing, China).

The recombinant plasmid, named as pET28a-*AlgVR7*, was transformed into *E. coli* BL21 (DE3) on Luria–Bertani (LB) media with 100 µg/mL Kanamycin. Single colonies of transformants were picked and verified through sequencing. The correct transformants carrying the pET28a-*AlgVR7* were incubated in an LB medium containing Kanamycin (100 µg/mL) at 37 °C until optical density 600 nm (OD_600_) reached 0.6–0.8. In order to induce the expression of *AlgVR7*, 0.5 mM isopropyl-β-D-thiogalactoside (IPTG) was added, and the incubation was continued for 16–18 h at 16 °C. The cells were collected via centrifugation and subsequently suspended in lysis buffer (50 mM PBS, pH 8.0), and then subjected to sonication.

The supernatant of cell lysis was purified through Ni–NTA resin (GE Healthcare, Stanford, CA, USA), equilibrated with binding buffer (50 mM PBS, 300 mM NaCl, pH 8.0). The other needless protein was washed off with washing buffer (50 mM NaH_2_PO_4_, 300 mM NaCl, 20 mM imidazole, pH 8.0), and the recombinant *AlgVR7* was eluted with elution buffer (50 mMNaH_2_PO_4_, 300 mM NaCl, 250 mM imidazole, pH 8.0). The *AlgVR7* was further analyzed using sodium dodecyl sulfate polyacrylamide gel electrophoresis (SDS-PAGE). Protein concentrations were determined using a BCA protein quantitative analysis kit (Beyotime Biotechnology, Shanghai, China).

### 4.4. Enzymatic Activity Assay

The amount of *AlgVR7* was controlled as 5 μg in one reaction mixture for characterization of the enzyme properties. The activity of *AlgVR7* was measured by the increase in absorbance at 235 nm (A_235_) due to the formation of the unsaturated saccharide. One unit was defined as an increase of 0.1 in A_235_ per min. The enzyme activity of *AlgVR7* was measured under different reaction conditions, including different temperatures (30–70 °C) and pH (4.0–10.0). The pH was set to be 8.0 to evaluate the effect of temperature on enzyme activity, and the temperature was kept at 40 °C to examine the effect of pH on enzyme activity. The thermostability of *AlgVR7* was tested by measuring the residual activities after exposure to 30, 40, 50, or 60 °C for 10–60 min followed by flash-cooling on ice. The pH stability of *AlgVR7* was studied by measuring the residual activities after incubation in different pH buffers (4.0–10.0) at 4 °C for 1 h. The residual enzyme activity was measured by the standard method. The enzyme activity without treatment was defined as 100%. The kinetic parameters of the purified enzyme toward these three kinds of substrates were determined by measuring the enzyme activity with substrates at different concentrations (0.1–10.0 mg/mL). The kinetic parameter values were calculated by using linear regression plots of Lineweaver and Burk [15].

### 4.5. Effects of Additives on Enzyme Stability

The *AlgVR7* was incubated with different additives at 40 °C for 1 h, and the residual enzyme activities were measured to examine their effects on *AlgVR7*’s stability under the most suitable conditions. The additives at 1 mM included various metal ions such as Na^+^, K^+^, Ca^2+^, Mg^2+^, Fe^2+^, Cu^2+^, Co^2+^, Mn^2+^, Ba^2+^, Cd^2+^, and Zn^2+^. The stability of *AlgVR7* was evaluated by exposing the enzyme to a high concentration of NaCl (0–500 mM) at 40 °C for 1 h. The enzyme activities for the treatments were normalized to the control without any addition (100%).

### 4.6. Substrate Specificity

The *AlgVR7* was incubated with different substrates to determine its substrate specificity, including polyM, polyG, sodium alginate, flaxseed gum, pectin, and polyglycolide acid (PGA). The reaction was initiated by adding 20 μL of enzyme solution (0.5 μg/mL) to 200 μL of 50 mM sodium phosphate buffer (pH 8.0) containing 2.0 mg/mL individual substrate. After incubation at 40 °C for 20 min, the assay of enzyme activity was defined as described previously.

### 4.7. ESI-MS Analysis of AlgVR7 Degradation Products

In order to determine the composition of the degradation products, 2 μL supernatant was loop-injected to an LTQ XL linear ion trap mass spectrometer (Thermo Fisher Scientific, Waltham, MA, USA). The oligosaccharides were detected in a positive-ion mode using the following settings: ion source voltage, 4.5 kV; capillary temperature, 275–300 °C; tube lens, 250 V; sheath gas, 30 arbitrary units (AU); scanning the mass range, 150–2000 *m*/*z*.

### 4.8. Molecular Docking

The overall structure of *AlgVR7* was predicted with AlphaFold2. Molecular docking was conducted using AutoDock Vina software. Using *AlgVR7* as a receptor and PG, PM, PGA, and PGA (DP3–DP8) as ligands, the binding energy was calculated after molecular docking, with lower binding energy values typically signifying a more robust connection between the ligand and protein. Hydrogen bonds and charges were added to the ligand and receptor, and the grid box was carefully assigned before the program was operated. PyMOL (DeLano Scientific LLC., San Francisco, CA, USA) and Discovery Studio 2019 (BIOVIA, San Francisco, CA, USA) were used to visualize and analyze the enzyme structure.

## 5. Conclusions

In conclusion, this study characterized *AlgVR7*, a bifunctional alginate lyase from *Vibrio* sp., which exhibits high catalytic efficiency and affinity towards various alginate substrates, including polyM, polyG, and PGA blocks. *AlgVR7*’s optimum temperature is 40 °C and its maximum activity occurs at pH 8, indicating its mesophilic, alkaline properties. Furthermore, *AlgVR7* demonstrates halophilic characteristics, exhibiting high stability in the presence of Na^+^ ions. The study of *AlgVR7*’s interactions with substrates revealed key amino acids and a unique loop1 associated with its catalytic activity. The phylogenetic analysis classified *AlgVR7* as a member of the PL7_5 subfamily with Glu167 proposed as a hallmark for the subfamily. Our findings highlight the potential applications of *AlgVR7* in biotechnology and industrial fields, particularly in the preparation of alginate oligosaccharides (AOS) that possess unique biological activities and health benefits. The insights gained from this research lay the foundation for future work on *AlgVR7* and have broader implications for understanding and utilizing alginate lyases from marine microorganisms.

## Figures and Tables

**Table 1:** Specific activities and kinetic parameters of *AlgVR7* toward sodium alginate, polyM, and polyG.

Substrate	Sodium Alginate	polyM	polyG
Specific activity (U/mg)	6854.6	3893.6	3845.0
*K*_m_ (μmol)	16.6	13.1	22.8
*V*_max_ (μmol/s)	186.8	150.9	176.8
*K*_cat_ (s^−1^)	16.9	13.7	11.4
*K*_cat_/*K*_m_ (s^−1^/μmol)	1.0	1.1	0.5

**Table 2:** Summarization of the reported bifunctional alginate lyases from *Vibrio* sp.

Organism	Enzyme	Optimal pH/Temperature (°C)	Substrate Specificity (U/mg)	*K*_m_/*V*_max_/*K*_cat_ (Alginate, polyM, and polyG)	Reference
*Vibrio rumoiensis* 402	*AlgVR7*	8.0/40	6854.6	*K*_m_ = 16.6, 13.1, 22.8 μmol *V*_max_ = 186.8, 150.9, 176.8 μmol/s *K*_cat_ = 16.9, 13.7, 11.4 s^−1^	This study
*Vibrio pelagius WXL662*	VpAly-I	6.0/40	194.0	N.D	[16]
*V. furnissii* H1	AlyH1	7.5/40	2.40 *	*K*_m_ = 2.28 mg/mL *V*_max_ = 2.81 U/mg toward alginate	[17]
*Vibrio* sp. QY101	AlyVI	7.5/40	N.D	*K*_m_ = 0.2223, N.D, 0.3274 mg/mL *V*_max_ = 3.6, N.D, 2.8321 U/mg	[18]
*Vibrio* sp. NJ-04	AlgNJ-04	7.0/30	2416	*K*_m_ = 0.49, 0.86, 0.24 mM *V*_max_ = 72, 95, 35 pmol/s; *K*_cat_ = 59, 77, 29 s^−1^	[10]
*Vibrio* sp. NJU-03	AlgNJU-03	7.0/30	6468.9	*K*_m_ = 8.50, 10.94, 4.00 mM *V*_max_ = 1.67, 0.30, 2.50 nmol/s	[19]
*Vibrio* sp. SY08	AlySY08	7.5/40	1070	N.D	[20]

Note: “*” represents the amount of reducing sugar released using the 3,5-dinitrosalicylic acid (DNS) method to determine alginate lyase activity. The enzyme activity measurement method not marked with “*” is the 235 nm absorbance method, and one unit is defined as the amount of enzyme required to increase the absorbance at 235 nm by 0.1 per min.

## Data Availability

The original data presented in the study are included in the article; further inquiries can be directed to the corresponding author.
